# Get Close to the Robot: The Effect of Risk Perception of COVID-19 Pandemic on Customer–Robot Engagement

**DOI:** 10.3390/ijerph18126314

**Published:** 2021-06-10

**Authors:** Jifei Wu, Xiangyun Zhang, Yimin Zhu, Grace Fang Yu-Buck

**Affiliations:** 1 School of Business, Sun Yat-Sen University, No. 135, Xingang Xi Road, Guangzhou 510275, China; wujf8@mail2.sysu.edu.cn (J.W.); zhangxy279@mail3.sysu.edu.cn (X.Z.); 2 School of Business and Technology, University of Maryland Eastern Shore, Princess Anne, MD 21853, USA; fyu@umes.edu

**Keywords:** COVID-19 pandemic, service robot, risk perception, customer engagement, protection motivation theory, social distancing

## Abstract

The purpose of this study was to examine the effect of the COVID-19 pandemic on customer–robot engagement in the Chinese hospitality industry. Analysis of a sample of 589 customers using service robots demonstrated that the perceived risk of COVID-19 has a positive influence on customer–robot engagement. The positive effect is mediated by social distancing and moderated by attitudes towards risk. Specifically, the mediating effect of social distancing between the perceived risk of COVID-19 and customer–robot engagement is stronger for risk-avoiding (vs. risk-seeking) customers. Our results provide insights for hotels when they employ service robots to cope with the shock of COVID-19 pandemic.

## 1. Introduction

The coronavirus disease 2019 (COVID-19) quickly became a global health emergency in 2020. Over 119 million people have contracted COVID-19, with over 2,600,000 deaths by 14 March 2021 [1]. The COVID-19 pandemic threatens not only people’s physical and mental health [2,3] but also the global economy, particularly the hospitality industry [4,5,6]. In 2020, hotel revenue fell by nearly 50% to $84.6 billion across the United States (US) [7], the largest since the Great Depression in 1933 [8]. It is estimated that it will take five years for the US hotel industry to recover to the same level of occupancy, average daily rates, and revenue as pre-COVID-19 times [9]. Therefore, it is crucial to understand how the pandemic has and may reshape customer behavior during and after the COVID-19 pandemic [8,10,11]. During the COVID-19 pandemic, many customers changed their behavior to maintain social distancing and reduce unnecessary social contact [12]. Further, the shift in customer views regarding social contact may fundamentally change the attitudes toward and the demand for service robots without human contact in the hospitality industry [10]. According to the latest report, the sales of service robots increased 24% in 2020, which will keep increasing in the future [13]. To clarify for the rest of the paper, ‘service robot(s)’ is defined as systems that function as programmable tools that can sense, think, and act to enhance human productivity or engage in social interactions [14,15].

In order to meet this shift in consumer demands, some hotels began to offer contactless services, such as service robots, to replace the frontline staff or allow guests not to have to interact with frontline staff [8,14,15]. For example, Hilton and Marriott hotels across California introduced service robots to provide services, such as delivering baggage and cleaning rooms [16]. Similarly, some restaurants now employ robots to take on some traditionally human work, including ordering, cooking, and delivering dishes [17]. During the COVID-19 pandemic, employing service robots reduces the possibility of transmitting the virus, which will also help service firms improve efficiency and decrease costs [8,18].

Prior research mostly focused on the general service scenario and documented reactance against service robots and other autonomous technologies [19,20,21,22,23]. For instance, customers preferred human labor over robot labor in the case of services or products with higher symbolic value [19] because human (vs. robotic) labor helps consumers meet unique needs. There are some studies showing that people will express a preference in specific contexts [24,25]. For example, people tend to rely on robots in objective decision-making tasks [24,25]. In the public health emergency of COVID-19, the perceived risk of customers has attached importance to academia and industry [4,5]. Prior qualitative studies explored how perceived risk is one of the key antecedents in many customer decisions. However, there was little research focus, at least from a quantitative perspective, on perceived risk and customer decision-making during a public health emergency. Therefore, we examined the impact of the perceived risk of COVID-19 on customer–robot engagement in a quantitative way.

Protection motivation theory [26,27] posits that individuals will estimate the level of threat. They will further build coping strategies when exposed to threat information related to health with their protective motivations. Accordingly, we draw on protection motivation theory [26,27,28] to understand whether and how the perceived risk of the COVID-19 pandemic influences customer engagement with service robots. We specifically investigate social distancing as the mediator of the relationship between customers’ perceived risk for the COVID-19 pandemic and customer–robot engagement. Finally, we explore the moderating effect of attitudes towards risk and health consciousness on the relationship between perceived risk of COVID-19 and customer engagement with service robots.

This study offers the following contributions. First, we employ protection motivation theory [26,27,28] to understand how the perceived risk of the COVID-19 pandemic influences consumer engagement with service robots. Previous research demonstrates that customers show negative attitudes to service robots in general service contexts [19,20,21,22,23]. This study finds that customers will be more engaged with service robots in public health emergencies, especially in the global pandemic of COVID-19. This study also aims to broaden the theoretical lens with regard to service robots and further expands the application scope of protection motivation theory. Second, following protection motivation theory [26,27], we explore the underlying mechanism of the relationship between the perceived risk of COVID-19 and customer engagement with service robots. The perceived risk of COVID-19 has a positive impact on social distancing and further influences customer engagement with service robots, which makes a contribution to understanding customer engagement with service robots in a public health emergency. Third, we clarify the boundary conditions of the indirect effect of COVID-19 on customer engagement with service robots. In particular, the mediating effect of social distancing between the perceived risk of COVID-19 and customer engagement with service robots is stronger for risk-avoiding customers compared to risk-seeking customers.

In the following sections, we first review the literature on service robots to develop our hypotheses. Next, we conduct a survey to test these hypotheses. Finally, we discuss theoretical contributions and managerial implications and conclude with limitations and future research directions.

## 2. Theoretical Framework

### 2.1. Perceived Risk of COVID-19 and Customer–Robot Engagement

Perceived risk is a variable connected to the probability and magnitude of the occurrence of the damage [29], which has been widely used to explain consumer behavior [8,30]. Consumer behavior researchers define perceived risk in terms of uncertainty and consequences. Perceived risk increases with higher levels of uncertainty and/or the chance of greater associated negative consequences [31,32]. For example, if a consumer is considering choosing an unfamiliar restaurant for a dinner party, the perceived risk associated with this choice could arise because he or she does not know how the dishes of the restaurant will taste (uncertainty) and is worried that guests will think poorly of him or her if it is not a good restaurant (negative consequences). In this study, we defined the perceived risk of COVID-19 as the possibility and the consequences as COVID-19 causing illness or death [32,33,34].

Following protection motivation theory [26,27], we propose that perceived risk triggered by COVID-19 will improve customer–robot engagement. When customers perceive the COVID-19 pandemic is riskier, they will perceive higher levels of uncertainty and infection [34,35]. Human beings are often regarded as the natural carriers of COVID-19 transmission. To reduce the risk of COVID-19, customers tend to be more avoidant of social contact with human staff than in normal times and attempt to social distance in restaurants and hotels. Indeed, choosing to engage with a service robot means a kind of avoidance to human frontline staff, which is viewed as a protection from being infected with COVID-19 [8,27,28].

Service robots can function as programmable tools which can sense, think, and act to engage in social interactions [14,15,36,37]. Prior research mostly focused on the context of general service, and this research documented reactance against service robots and autonomous technologies [19,20,21,22,23]. However, little focus has been on situations where customers would possibly prefer service robots and would choose to engage with a service robot [24,25]. It is necessary to explore the antecedents for consumers to engage with service robots and the underlying psychological mechanisms.

As a way to build and strengthen customer relationships, customer engagement can help companies establish a competitive advantage and achieve success [38]. In addition, it can improve customer satisfaction, customer loyalty, and company performance [38,39,40]. Customer engagement is a multi-dimensional concept, including cognitive, emotional, and behavioral aspects [39,41,42,43]. Previous research has focused on customer engagement with brand [38,40], community [41], organization [44], and other traditional objectives in marketing practice.

In the COVID-19 pandemic, there are more service robots employed in hotels and restaurants [8,15], and customers have begun to engage with these robots. Thus, we introduce the concept of customer engagement in the context of service robots. We define customer engagement with service robots (hereafter, customer–robot engagement) as the customer’s personal connection to service robots that goes beyond transactions, including the reaction in cognition, emotion, and behavior [45]. Customer–robot engagement in the context of hospitality consists of attention, enthusiasm, and interaction. Attention describes the extent of customer paying attention to the service robot [46,47]; enthusiasm means how much customers are interested in and excited to be serviced by the robot [44,48]; interaction points out that customers share service robots with others or participate in online and offline activities related to a service robot [44,48,49].

In line with protection motivation theory [26,27,50], we explore the effect of the perceived risk of COVID-19 on customer–robot engagement in the hotel and restaurant industries. Protection motivation theory is a social cognitive theory that was developed to explain the influences of health threats on health attitudes and behaviors [26,27,28,50]. According to protection motivation theory, threat appraisal and coping appraisal are the two primary drivers of health behavior [26,27]. Threat appraisal refers to the beliefs about the severity and susceptibility of the health threat to the given person, which concerns the health threat’s nature, its seriousness, and the propensity of it eventuating to affect the individual [26,34]. Coping appraisal refers to the evaluation of health-protective behavioral alternatives and responses to avoid the health threat and the negative consequences, which focuses on the effectiveness of the coping response to impede the threat [26,27,51].

When risk is salient, customers will show preference to a hotel with a service robot staff than a hotel with human staff [52]. Thus, when perceived risk is higher, the motivation is stronger to cope with uncertainty and the subsequent consequences [26,27,50]. Further, customers will be more likely to engage with the service robots. Specifically, customers will pay more attention to the service robots, will show more enthusiasm to the service robots, and will have more interactions with the service robots.

In sum, it is expected that customers who perceive a high level of risk for COVID-19 are more likely to engage with service robots in restaurant and hospitality services. Therefore, we propose the following hypotheses:

**Hypothesis** **1a** **(H1a).** *Perceived risk of COVID-19 has a positive influence on customer–robot engagement, i.e., customers’ attention to service robots*.

**Hypothesis** **1b** **(H1b).** *Perceived risk of COVID-19 has a positive influence on customer–robot engagement, i.e., customers’ enthusiasm in service robots*.

**Hypothesis** **1c** **(H1c).** *Perceived risk of COVID-19 has a positive influence on customer–robot engagement, i.e., customers’ interaction with service robots*.

### 2.2. The Mediating Role of Social Distancing

According to protection motivation theory, a higher perceived health risk will lead customers to take measures to avoid risks and protect themselves [28,51]. For example, consumers will reduce some purchase behaviors, which may bring negative consequences [53]. They will become more conservative, keeping their distance from new or risky products and services [30]. In addition, they will avoid negative consequences and take measures to protect themselves. In the COVID-19 pandemic, social distancing is a crucial measure to protect consumers when they perceive the risk of transmission of COVID-19.

Many governments promoted the prevention policies of quarantining or social distancing (i.e., maintaining a physical distance of at least 2 m (6 feet)) [54]. It is hard to keep this precise distance for most customers. A number of customers choose to reduce social contacts in order to maintain social distance and to comply with the government’s prevention policy. Furthermore, many consumers have reduced their international travel and have cut down on other journeys to areas with large COVID outbreaks. In the COVID-19 pandemic, social distancing is considered an effective coping response to impede the COVID-19 threat [8,10,54]. For this reason, customers will be more willing to socially distance as a kind of protective or coping method in service places when they perceive a higher risk of COVID-19. Once they perceive higher health risks, they will be active in protective behaviors [26,51], including social distancing. Even after quarantine, many customers continued to engage in avoidance and protective behaviors in service places [55]. Therefore, we infer that the perceived risk of COVID-19 will influence customer social distancing.

If customers want to keep social distancing, they will likely embrace some options that would reduce social contact [52]. Once the intention of keeping social distancing was increased, people would decrease direct contact with humans [12], and they will be more likely to engage with services provided by robots. Even service robots can convey social meaning to customers; they are mostly functional service robots, which perform labor such as ordering or delivery in hotels and restaurants. Engagement with service robots can replace some social activities and reduce risk from social contact. Engagement with a service robot can be viewed as a protective method, which can reduce the chances of being infected with COVID-19. In addition, service robots can interact with humans, replacing some social activities [23]. Based on these functions of service robots, service robots can be an attractive consumer choice to protect themselves in the context of a public health emergency. Specifically, customers will pay more attention to service robots, show more enthusiasm to service robots, and seek out more interactions with service robots. As a result, we propose the following hypotheses:

**Hypothesis** **2a** **(H2a).** *The influence of perceived risk of COVID-19 on customer–robot engagement, i.e., customers’ attention to service robots*.

**Hypothesis** **2b** **(H2b).** *The influence of perceived risk of COVID-19 on customer–robot engagement, i.e., customers’ enthusiasm in service robots*.

**Hypothesis** **2c** **(H2c).** *The influence of perceived risk of COVID-19 on customer–robot engagement, i.e., customers’ interaction with service robots, is mediated by the social distancing*.

### 2.3. The Moderating Role of Risk Attitude

Risk attitude can reflect a decision-maker’s intention to take risk or to avoid risk [56]. There are two types of attitudes towards risk: risk-seeking and risk-avoiding. Because many decisions are generally made under a certain level of risk, the optimal choice from a decision-maker’s perspective will depend on their attitude towards risk [56,57]. Risk attitude has a wide-ranging influence on many types of behaviors, including trading behavior, unhealthy behavior, and work practice [58,59,60]. In this paper, we propose that attitude towards risk moderates the mediating effect of social distancing.

Due to the individual differences in risk attitude, some are motivated by the upside potential of risk, while others are motivated by security [61]. For risk seekers, perceived risk will not hinder their subsequent behaviors in some choices, including investment decisions and treatment choices [56,59,62]. So, risk seekers will pay less attention to service robots, show less enthusiasm to service robots, and will have fewer interactions with service robots when they perceive a high risk of COVID-19. But for the risk-averse, coping with risk is emphasized. And risk-averse individuals are less likely to engage in risky or unhealthy behavior, such as smoking and drug use [58]. If the perceived risk of COVID-19 is large, risk-averse consumers will engage in protective behavior to avoid infection, leading to social distancing and more customer–robot engagement. Thus, we propose that the positive effect of the perceived risk of COVID-19 on social distancing is stronger for risk-averse (vs. risk-seeking) customers.

**Hypothesis** **3a** **(H3a).** *The mediating effect of social distancing on the relationship between the perceived risk of COVID-19 and customer–robot engagement, i.e., customers’ attention to service robots*.

**Hypothesis** **3b** **(H3b).** *The mediating effect of social distancing on the relationship between the perceived risk of COVID-19 and customer–robot engagement, i.e., customers’ enthusiasm in service robots*.

**Hypothesis** **3c** **(H3c).** *The mediating effect of social distancing on the relationship between the perceived risk of COVID-19 and customer–robot engagement, i.e., customers’ interaction with service robots, is stronger for risk-averse (vs. seeking) customers*.

### 2.4. The Moderating Role of Health Consciousness

Health consciousness is defined as the tendency to focus on one’s health [63]. Health-conscious consumers are more concerned about their health. They strive to enhance and/or sustain their healthy state by engaging in healthy behaviors [64]. Health consciousness fosters preventive health care, positive attitudes towards healthy behaviors, and purchases of health-related products [65,66,67]. Individuals will react to health risks differently depending on their level of health consciousness [68]. We propose that health consciousness will moderate the mediating effect of social distancing.

Health consciousness greatly impacts how people respond to health-related messages [63]. Health-conscious consumers will pay much more attention to coping with the risk related to health [64]. Researchers report a positive correlation between health consciousness and the tendency to engage in preventive health behaviors [65]. If people with high health consciousness perceive a higher level of health risk from COVID-19, they will keep social distancing and will be more likely to engage with robots. In contrast, for consumers who are not health-conscious, the effect of perceived risk on social distancing is reduced. They will also not pay more attention to service robots, they will show less enthusiasm to service robots, and they will have fewer interactions with service robots. Thus, we propose:

**Hypothesis** **4a** **(H4a).** *The mediating effect of social distancing on the relationship between perceived risk of COVID-19 and customer–robot engagement, i.e., customers’ attention to service robots*.

**Hypothesis** **4b** **(H4b).** *The mediating effect of social distancing on the relationship between perceived risk of COVID-19 and customer–robot engagement, i.e., customers’ enthusiasm in service robots.*

**Hypothesis** **4c** **(H4c).** *The mediating effect of social distancing on the relationship between perceived risk of COVID-19 and customer–robot engagement, i.e., customers’ interaction with service robots, is stronger for high (vs. low) health consciousness customers*.

**Figure 1:**
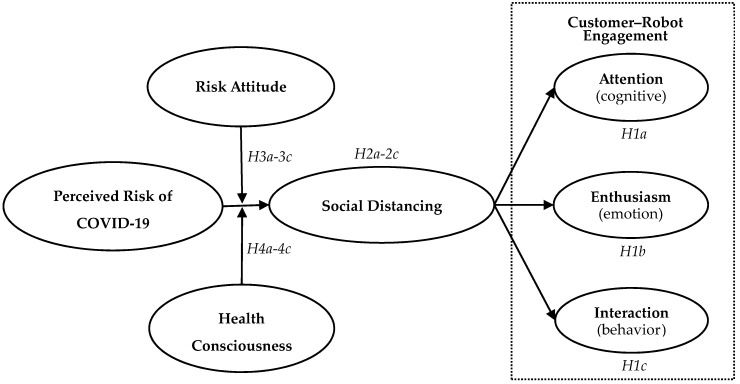
Conceptual model.

In sum, the proposed model is summarized in Figure 1.

## 3. Methodology

### 3.1. Sample

A survey was employed to collect data to test our hypotheses. We chose the customers in Chinese hospitality with service robots as our respondents. There are two reasons. First, the early outbreak of COVID-19 caused unprecedented damage to hospitality industries in China. Second, several service robots have been introduced into hotels and restaurants in China, which provide services, such as ordering and delivering dishes, without social contact.

All multi-item constructs with existing scales were adapted from the public health, marketing, and tourism literature. Validity and reliability were ensured by back-translating the measures. Before our formal survey, we invited three professors and three Ph.D. students to examine our items. Based on their advice, we revised the items and kept the language of the items clear, specific, and simple. We also conducted a pretest and collected 53 surveys. Factor analysis was used to test the reliability and validity of the measurements to ensure the effectiveness of the follow-up survey further.

In order to guarantee the confidentiality and quality of data, we invited our respondents randomly who received service from service robots in the hospitality industry. Every responder spent about 4 min answering this survey. All respondents received RMB 4 as the payment for participating in our survey.

We invited the respondents randomly to participate in our survey through Wenjuanxing (www.wjx.cn) (accessed on 1 September 2020), the biggest survey platform in China. A total of 647 customers were invited from September to October 2020, when the outbreak of COVID-19 was largely controlled in China. In total, 36 respondents were removed because of failing to pass the attention tests or taking an unreasonably short time (i.e., less than two minutes), and 22 respondents were discarded because of incomplete data (>25% of answers omitted). In total, 589 valid respondents were used for our data analyses. The demographic profile of the sample is shown in Table 1. Approximately 51.4% of respondents were female, whereas 48.6% were male. The majority of respondents were aged 18 to 39 (97.1%) and had a bachelor’s degree (68.8%). In addition, a plurality (38.5%) of respondents had yearly income between 5000 and 100,000 RMB. The second most common was an income between 10,000 and 20,000 RMB (24.7%), and third most common was less than 5000 RMB (20%).

### 3.2. Measures

We measured all multi-item constructs with existing scales drawn from the tourism, marketing, and healthcare literature (Table 2), using a seven-point Likert format (1 = strongly disagree/not at all; 7 = strongly agree/extremely) for all measures except attitude towards risk. Specifically, the perceived risk of COVID-19 was evaluated by two items adopted from Kim and Lee [69] and Gidengil et al. [68]. Customer–robot engagement was measured in terms of attention (four items), enthusiasm (four items), and interaction (four items), and this methodology was adopted from So et al. [40]. Social distancing was assessed by two items adopted from Aron [70]. Health consciousness was evaluated by four items adopted from Gineikiene et al. [71].

We also measured risk attitude, which was assessed by five items adopted from Forlani and Mullins [56], i.e., please answer the following 5 items by circling the alternative (“a” or “b”) you would feel most comfortable with. 1. (a) an 80% chance of winning $400, or (b) receiving $320 for sure; 2. (a) receiving $300 for sure, or (b) a 20% chance of winning $1500; 3. (a) a 90% chance of winning $200, or (b) receiving $180 for sure; 4. (a) receiving $160 for sure, or (b) a 10% chance of winning $1600; 5. (a) a 50% chance of winning $500, or (b) receiving $250 for sure.

Finally, the technology adoption model (TAM) literature deems that the customer behavior related to new technology is influenced by customer-level factors regarding the perception of the technology, such as perceived usefulness and perceived ease of use [72,73,74]. Therefore, we controlled for these variables to minimize omitted variable bias and account for factors that explained significant variance in customer–robot engagement. We measured perceived usefulness (four items) and perceived ease of use (four items) with scales adapted from Davis [72] and Agarwal and Karahanna [75].

### 3.3. Data Analysis

The marker-variable technique [76] was employed to statistically identify the threat of common method variance (CMV). Confirmatory factor analysis (CFA) was performed to evaluate the reliability and validity, and structural equation modeling (SEM) was used to examine the direct hypotheses. The bootstrapping approach based on PROCESS macro [77] was used for the mediation analysis and moderation analysis. These data analyses were conducted using SPSS 24.0 (IBM, New York, NY, USA) and Amos 24.0 (IBM, New York, NY, USA).

## 4. Results

### 4.1. Reliability and Validity

Table 2 shows the results of the CFA. The CFA resulted in good fit to the data (χ^2^/df = 2.71, GFI = 0.904, NFI = 0.980, CFI = 0.987, RMSEA = 0.054). The composite reliability was satisfactory as well because the scores for all constructs ranged from 0.87 to 0.97, exceeding the threshold of 0.70 [78]. Our instrument demonstrated convergent validity, as all factor loadings were between 0.70 and 0.97, greater than the recommended minimum value of 0.50; the average variance extracted (AVE) for each construct ranged from 0.62 to 0.94, greater than the threshold of 0.50 [79].

The results in Table 3 indicated strong discriminant validity, as the square roots of the AVEs were greater than the corresponding correlation coefficients between the factors [80].

### 4.2. Common Method Biases

In addition to program control, statistical controls were employed to assess the common method biases [81]. We adopted the marker-variable technique [76] to evaluate the common method biases and took education level as a marker variable. As shown in Table 3, the correlation coefficients between education level and other variables were small and not significant (*p* > 0.05). Thus, the common method biases of the current study were not serious.

Consistent with Schwepker’s study [82], we used the CFA technique to analyze potential common method biases using three steps. First, all items point to the latent variables measured by them, and carry out an eight-factor model CFA, which is called model C1. Second, all items point to the common method biases variable and carry out a one-factor model CFA, which is called model C2. Third, we compared the changes of model fit indexes of model C1 and model C2 to see if a significant difference emerged. As shown in Table 4, the model fit of model C2 was poor, and the model fix of model C1 improved fit significantly (Δχ^2^ = 3735.12, Δdf = 28, *p* < 0.001), which means that the common method biases were not serious.

### 4.3. Hypotheses Test

#### 4.3.1. Direct Effect Analysis

**Figure 2:**
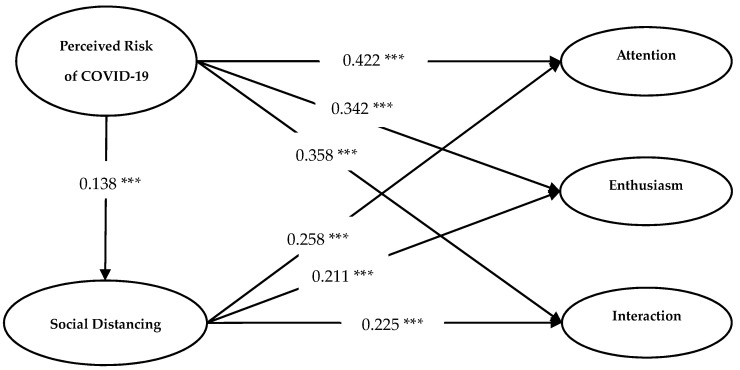
Results of the SEM. *** *p* < 0.001.

Figure 2 displays the results of the SEM. The fit indices (χ^2^/df = 2.81, GFI = 0.921, NFI = 0.945, CFI = 0.958, RMSEA = 0.073) indicated the appropriateness of the structural model [83]. The path coefficients from perceived risk of COVID-19 to customers’ attention (β = 0.422, *p* < 0.001), enthusiasm (β = 0.342, *p* < 0.001), and interaction (β = 0.358, *p* < 0.001) were positively significant. Therefore, H1a, H1b and H1c, which proposed that perceived risk of COVID-19 had positive influences on customer–robot engagement (respectively, attention, enthusiasm, and interaction), were supported.

#### 4.3.2. Mediation Analysis

The bootstrapping procedure suggested by Hayes [77], with a confidence level of 95% and a bootstrap sample of 5000, was conducted to examine the mediating effect of social distancing. The analysis results are shown in Table 5. All the concerned 95% confidence intervals excluded the value of 0, thereby supporting the indirect effects of perceived risk on customers’ attention (effect size = 0.088, SE = 0.026, 95% CI [0.045, 0.147]), enthusiasm (effect size = 0.078, SE = 0.021, 95% CI [0.043, 0.126]) and interaction (effect size = 0.099, SE = 0.024, 95% CI [0.059, 0.151]) through social distancing. These results implied social distancing mediated the effect of perceived risk on customer–robot engagement. Therefore, H2a, H2b, and H2c were supported.

#### 4.3.3. Moderated Mediation Analysis

The bootstrapping procedure based on PROCESS macro suggested by Hayes [77], with a confidence level of 95% and a bootstrap sample of 5000, was conducted to examine H3a to H4c. The analysis results are shown in Table 6.

**Figure 3:**
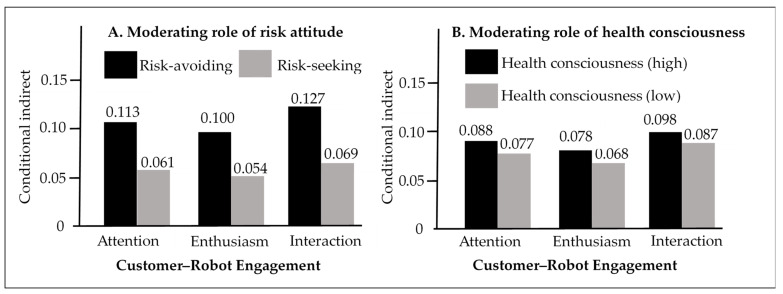
Conditional indirect effect. (**A**) Moderating role of risk attitude. (**B**) Moderating role of health consciousness.

Using attitude towards risk as the moderator, the index of moderated mediation was significant for customers’ attention (index = 0.052, SE = 0.020, 95% CI [0.018, 0.098]), enthusiasm (index = 0.046, SE = 0.016, 95% CI [0.016, 0.085]), and interaction (index = 0.059, SE = 0.022, 95% CI [0.019, 0.104]), indicating the risk attitude moderated the mediating effects of social distancing on the relationship between perceived risk and customers’ attention, enthusiasm, and interaction. For risk-averse consumers, social distancing significantly mediated the effect of perceived risk on customers’ attention (effect size = 0.113, SE = 0.032, 95% CI [0.060, 0.182]), enthusiasm (effect size = 0.100, SE = 0.025, 95% CI [0.057, 0.156]), and interaction (effect size = 0.127, SE = 0.029, 95% CI [0.078, 0.189]). In contrast, for risk-seeking customers, the mediating effect of social distancing on customers’ attention (effect size = 0.061, SE = 0.026, 95% CI [0.022, 0.125]), enthusiasm (effect size = 0.054, SE = 0.032, 95% CI [0.060, 0.182]), and interaction (effect size = 0.069, SE = 0.025, 95% CI [0.029, 0.128]) were still significant but the effect sizes were considerably reduced (attention: from 0.113 to 0.061; enthusiasm: from 0.100 to 0.054; interaction: from 0.127 to 0.069, Figure 3A), in support of H3a, H3b, and H3c.

Using health consciousness as the moderator, the index of moderated mediation was not significant for customers’ attention (index = 0.006, SE = 0.010, 95% CI [−0.013, 0.025]), enthusiasm (index = 0.005, SE = 0.008, 95% CI [−0.012, 0.021]), and interaction (index = 0.007, SE = 0.011, 95% CI [−0.015, 0.027]), which means the mediating effect sizes were not significant difference between high and low levels of health consciousness (Figure 3B). Specifically, for high levels of health consciousness, social distancing significantly mediated the effect of perceived risk on customers’ attention (effect size = 0.088, SE = 0.026, 95% CI [0.050, 0.149]), enthusiasm (effect size = 0.078, SE = 0.020, 95% CI [0.045, 0.126]), and interaction (effect size = 0.098, SE = 0.023, 95% CI [0.060, 0.152]). Similarly, for low levels of health consciousness, the mediating effect of social distancing on customers’ attention (effect size = 0.077, SE = 0.027, 95% CI [0.035, 0.139]), enthusiasm (effect size = 0.068, SE = 0.022, 95% CI [0.033, 0.119]), and interaction (effect size = 0.087, SE = 0.026, 95% CI [0.045, 0.143]) were still significant. There was no significant difference between high and low levels of health consciousness (attention: from 0.088 to 0.077; enthusiasm: from 0.078 to 0.068; interaction: from 0.098 to 0.087, Figure 3B). These results showed that the health consciousness did not moderate the mediating effects of social distancing on the relationship between perceived risk and customer–robot engagement. Thus, H4a, H4b, and H4c were not supported.

## 5. General Discussion

### 5.1. Theoretical Implications

These findings of this paper have three theoretical contributions. First, most previous research suggested that people have a negative attitude toward service robots in the general service context [19,20,21,22,23]. In addition, prior research lacks the discussion of the role of perceived risk during the COVID-19 pandemic from a quantitative aspect [4,5,6,7,8]. This study focuses on the effect of the perceived risk of COVID-19 on customer–robot engagement in a public health emergency, which expands the perspective of research on service robots. Prior research on customer engagement has mostly discussed customer engagement with brand, product, and community [38,40,42,44,45]. In addition, there is some research arguing that anthropomorphism increases the intention of the customer to be close to other objects, including service robots [84,85]. The anthropomorphism of service robots provides another choice for social activities when there is a higher level of the perceived risk of COVID-19. This work discusses the impact of the perceived risk of a public health emergency on customer–robot engagement, which is rapidly developing and popular among hospitality industries. We find that the perceived risk in the COVID-19 pandemic can increase customer–robot engagement significantly, which extends the research on the antecedents of customer engagement. And the results of this study enrich the research of anthropomorphism and service robots as it replaces some human staff in hotels and restaurants.

Second, we are the first to utilize protection motivation theory [26,50] to explain how customers’ perceived risk of the COVID-19 pandemic influences customer–robot engagement. Our results showed that the perceived risk of COVID-19 positively influences customer–robot engagement through the influence of social distancing, which helps deepen understanding of customer–robot engagement in a public health emergency. In line with protection motivation theory [26,50], this work demonstrated that social distancing is a critical form of coping strategy when faced with the risk of COVID-19. This research emphasized the importance of social distancing in coping with COVID-19 [12].

Third, we discussed the moderators of the indirect effect of COVID-19 on customer–robot engagement. In particular, the mediating effect of social distancing on the relationship between perceived risk of COVID-19 and customer–robot engagement is stronger for risk-averse (vs. risk-seeking) customers. This work enriches the knowledge of coping strategies for COVID-19, and it offers a new context to improve and innovate robot services.

### 5.2. Practical Implications

This study has important implications for how to utilize service robots to cope with a public health emergency. First, we provide some advice as to whether a company should introduce service robots into frontline service. For managers in the hospitality industry, it is one of the important decisions to employ service robots. The reason why many companies chose not to employ service robots without social contact is that previous research and reports have shown that customers have negative attitudes to service robots and other automation technologies [19,20,21,22,23]. The outbreak of COVID-19 had a large impact on hospitality industries whose business mainly depend on social contact. Our work found that customers tend to engage with service robots during the COVID-19 pandemic, which supports the decision for managers to introduce service robots into their hotels and restaurants. Indeed, employing service robots may help companies improve performance, reduce the risk of infection for human staff, and maintain customer relationships during the COVID-19 pandemic.

Second, this work explored the motivation of customer–robot engagement in the pandemic. When people are exposed to health threat information, their protection motivation will be enhanced [26,50], and they will increase customer–robot engagement. Thus, we suggest that hotels and restaurants should employ service robots effectively based on the protection motivation of customers. For instance, companies can emphasize the security of a service robot to cater to the customers’ need for protection during and after the COVID-19 pandemic. In addition, this paper showed that social distancing is a mediator in the relationship between perceived risk and customer–robot engagement. Accordingly, hotels and restaurants may encourage, via promotional campaigns, customers to accept service from robot staff to maintain social distancing better.

Third, some references have been provided by this research for companies that aim to improve customer engagement with advanced technologies. Customer engagement can develop and strengthen customer relationships, and it can enhance customer loyalty and company performance. We demonstrated the forms of customer–robot engagement, including attention, enthusiasm, and interaction [39,41,43]. The conclusion of this paper offers guidance for designing a customer engagement approach in the hospitality industry. For example, hotels and restaurants may develop campaigns that focus on customer experience with service robots to improve attention, enthusiasm, and interaction of customers. If customers are attracted to participate in campaigns to engage with service robots, customer loyalty and company performance will be improved.

### 5.3. Limitations and Future Research

There are limitations and opportunities for future research. First, there are many antecedents for customers to engage with service robots. However, this study focused on one key antecedent, the perceived risk of COVID-19, and its effect on customer–robot engagement. Other antecedents for customer–robot engagement should be explored in a follow-up study.

Second, we explored the impact of the perceived risk of COVID-19 on customer–robot engagement and the underlying mechanism. However, it is worth exploring whether this effect will shift after the COVID-19 pandemic. Future research may discuss the long-term influence of the public health emergency through collecting data after the COVID-19 pandemic ends. In addition, the role of perception of social ability for service robots can be explored in the future. If customers can perceive more social closeness with service robots, they will have more intention to engage with service robots.

Finally, we deepened the understanding of the influence of the perceived risk of COVID-19 on customer–robot engagement based on protection motivation theory. And we discussed the mediation of social distancing. There are other possible theories or mechanisms to explain this influence. One future direction for research is the uncanny valley [86], which may explain the reason why customers choose to engage with a service robot or not. Moreover, the motivation of customer–robot engagement varies according to the service context. Future research may discuss customer engagement in other contexts.

## 6. Conclusions

Although previous research analyzed customer attitudes toward service robots in the general service context [37,87,88,89], little research has taken the context of public health emergencies into account. This research aimed to discuss the effect of perceived risk on customer–robot engagement in a public health emergency.

First, perceived risk has a positive impact on customer–robot engagement. Specifically, when the perceived risk of COVID-19 is at a higher level, there will be stronger protection motivation for customers. Further, customers will pay more attention to service robots, show more enthusiasm towards service robots, and have more interaction with service robots. Before the COVID-19 pandemic, most researchers found that customers preferred service to be provided by human staff rather than by service robots [37,87,88,89]. Some research on anthropomorphism argued that anthropomorphized robots could reduce resistance from customers [84,85]. Anthropomorphism of the robot is the attribution of human characteristics or behavior to a robot [84,85]. When customers perceived the risk of COVID-19 is at a higher level, customers have a tendency to reduce contact with human staff. Based on the anthropomorphism of service robots, people can contact service robots to replace some social activities. Service robots satisfy the social need of customers and take the place of human staff to some extent. Our findings suggest that due to the higher perceived risk of COVID-19, customers are more likely to engage with service robots in the pandemic. It demonstrates that COVID-19 may accelerate the process of acceptance of service robots without human contact, as service robots decrease the risk of COVID infection by allowing easier social distancing.

Second, our research showed that social distancing is the mediator of the effect of the perceived risk of COVID-19 on customer–robot engagement. When customers are faced with health threat information regarding COVID-19, they will appraise the health threat, including its severity and their vulnerability. When the perceived risk is high, customers will adopt a coping strategy and will strengthen social distancing, which will further enhance customer–robot engagement.

Third, risk attitude moderates the mediating effect of social distancing. The mediating effect of social distancing on the relationship between perceived risk of COVID-19 and customer–robot engagement is stronger for risk-averse (vs. risk-seeking) customers. Compared to risk-seeking customers, customers who are risk-averse attach more importance to the coping strategy of health risk. Thus, when they perceive a higher level of risk, their willingness to socially distance will be stronger, and their engagement with service robots will be enhanced.

Finally, our results showed that the moderating effect of health consciousness is not significant. The possible reason is that the direct effect of perceived risk diminishes the moderating effect of health consciousness. In the COVID-19 pandemic, customers with different levels of health consciousness perceive a high level of risk, and they would like to keep social distance and further choose to engage with service robots.

## Figures and Tables

**Table 1:** Demographic profile of the sample (*n* = 589).

Variable	Items	(%)
Gender	Male	48.6
	Female	51.4
Age	18–24	32.9
	25–29	35.5
	30–39	28.7
	40–56	2.9
Education level	High school degree	5.9
	Associate degree	11.7
	Bachelor’s degree	68.8
	Graduate degree	13.6
Income level	Under RMB 5000	20.0
	RMB 5001–10,000	38.5
	RMB 10,001–20,000	24.7
	RMB 20,001–50,000	11.7
	Over RMB 50,000	5.1

**Table 2:** Measured items and CFA results.

Variables and Items	Factor Loading	α	CR	AVE
**Perceived risk**	-	0.77	0.89	0.81
What are the chances of you getting infected with the COVID-19?	0.91			
What are the chances of you dying from the COVID-19 if infected?	0.89			
**Social distancing**	-	0.93	0.97	0.94
To what extent do you think you have an increased need to keep social distancing from others during the COVID-19?	0.97			
To what extent do you engage in social distancing during the COVID-19?	0.97			
**Customer engagement**				
***Attention***	-	0.90	0.93	0.76
I pay a lot of attention to service robots.	0.89			
I like to learn more about service robots.	0.89			
I like learning more about service robots.	0.88			
Anything related to service robots grabs my attention.	0.85			
***Enthusiasm***	-	0.89	0.92	0.75
I am passionate about service robots.	0.88			
I am enthusiastic about service robots.	0.90			
I feel excited about service robots.	0.87			
I love this service provided by robots.	0.83			
***Interaction***	-	0.87	0.91	0.72
In general, I like to get involved in service robot discussions.	0.87			
In general, I thoroughly enjoy exchanging ideas with other people about service robots.	0.86			
I often browse new topics about service robots.	0.85			
I often share my experience with service robots.	0.81			
**Perceived ease of use**	-	0.90	0.93	0.76
Learning to operate the robot is easy for me.	0.87			
I find it easy to get the robot to do what I want it to do.	0.85			
It is easy for me to become skillful at using the robot.	0.90			
I find the robot easy to use.	0.88			
**Perceived usefulness**	-	0.85	0.90	0.69
Using the robot enhances service effectiveness in the hotel.	0.80			
Using the robot enhances service productivity.	0.85			
I find the robot useful in hotel service.	0.84			
Using the robot improves service performance in hotels.	0.83			
**Health consciousness**	-	0.79	0.87	0.62
I reflect on my health a lot.	0.70			
I’m very self-conscious about my health.	0.80			
I am generally attentive to my inner feelings about my health.	0.84			
I am constantly examining my health.	0.80			

Notes. α, Cronbach’s α; CR, composite reliability; AVE, average variance extracted.

**Table 3:** Descriptive statistics and correlation matrix of variables.

Variables	1	2	3	4	5	6	7	8	9
1. Perceived risk	**0.90**								
2. Social distancing	0.48 **	**0.97**							
3. Attention	0.52 **	0.54 **	**0.87**						
4. Enthusiasm	0.51 **	0.52 **	0.81 **	**0.87**					
5. Interaction	0.55 **	0.58 **	0.85 **	0.80 **	**0.85**				
6. Perceived ease of use	0.28 **	0.35 **	0.48 **	0.41 **	0.47 **	**0.87**			
7. Perceived usefulness	0.31 **	0.33 **	0.52 **	0.597 **	0.54 **	0.46 **	**0.83**		
8. Health consciousness	0.33 **	0.37 **	0.47 **	0.469 **	0.49 **	0.41 **	0.40 **	**0.79**	
9. Education level	−0.05	0.00	0.04	0.02	0.02	0.02	0.03	0.02	-
Mean	5.53	5.52	5.63	5.84	5.63	5.51	6.04	5.85	2.90
SD	1.07	1.27	1.08	1.01	1.04	1.11	0.81	0.86	0.69

Note. The values in the lower diagonal of the table present the correlations between the constructs, while the values in the diagonal of the table present the square roots of the AVEs of the construct. We take education level as a marker variable 3. *n* = 589; ** *p* < 0.01. Bold: the square roots of the AVE for each construct.

**Table 4:** The CFA model fit.

Index	χ^2^	df	CFI	NFI	GFI	RMSEA
Model C1 (eight factors model)	871.85	322	0.987	0.980	0.904	0.054
Model C2 (one factor model)	4606.97	350	0.914	0.907	0.641	0.144
Δ = Model C2-Model C1	Δχ^2^ = 3735.12	Δdf = 28	*p* < 0.001			

**Table 5:** Mediating effect analysis results (*n* = 589).

Paths	Indirect Effect	LLCI	ULCI
Perceived risk → Social distancing → Attention	0.088	0.045	0.147
Perceived risk → Social distancing → Enthusiasm	0.078	0.043	0.126
Perceived risk → Social distancing → Interaction	0.099	0.059	0.151

Note. LL = Lower limit, UL = Upper limit, CI = Confidence interval. SE, standardized error. The value of the lower limit and that of the upper limit constitutes a confidence interval.

**Table 6:** Analysis results for the moderated mediation effect (*n* = 589).

DVs	Moderator	Indirect Effect of Social Distancing				Moderated Meditation Effect			
		Effect Size	SE	LLCI	ULCI	Index	SE	LLCI	ULCI
Attention	Risk attitude (seeking)	0.061	0.026	0.022	0.125	0.052	0.020	0.018	0.098
	Risk attitude (avoid)	0.113	0.032	0.060	0.182				
Enthusiasm	Risk attitude (seeking)	0.054	0.021	0.021	0.106	0.046	0.018	0.016	0.085
	Risk attitude (avoid)	0.100	0.025	0.057	0.156				
Interaction	Risk attitude (seeking)	0.069	0.025	0.029	0.128	0.059	0.022	0.019	0.104
	Risk attitude (avoid)	0.127	0.029	0.078	0.189				
Attention	Health consciousness (high)	0.088	0.026	0.050	0.149	0.006	0.010	−0.013	0.025
	Health consciousness (low)	0.077	0.027	0.035	0.139				
Enthusiasm	Health consciousness (high)	0.078	0.020	0.045	0.126	0.005	0.008	−0.012	0.021
	Health consciousness (low)	0.068	0.022	0.033	0.119				
Interaction	Health consciousness (high)	0.098	0.023	0.060	0.152	0.007	0.011	−0.015	0.027
	Health consciousness (low)	0.087	0.026	0.045	0.143				

Notes. DVs, dependent variables; SE, standardized error. Perceived risk as the independent variable, social distancing as the mediator, risk attitude, and health consciousness as moderators. Confidence interval (CI) was 95%. Bootstrap samples was 5000. Risk attitude: seeking = 0, avoiding = 1.

## Data Availability

The dataset used in this research are available upon request from the corresponding author. The data are not publicly available due to restrictions, i.e., privacy or ethical.
